# Efficacy and safety of blood derivatives therapy in Alzheimer’s disease: a systematic review and meta-analysis

**DOI:** 10.1186/s13643-022-02115-y

**Published:** 2022-11-29

**Authors:** Zhangcheng Fei, Bo Pan, Renjun Pei, Zhongsheng Chen, Xi Du, Haijun Cao, Changqing Li

**Affiliations:** Institute of Blood Transfusion, Chinese Academy of Medical Sciences and Peking Union Medical College, Chengdu, 610052 China

**Keywords:** Blood derivatives, Alzheimer’s disease, IVIG, Plasma exchange, Plasma infusion, Meta-analysis

## Abstract

**Background:**

Blood derivatives therapy is a conventional clinical treatment, while the treatment for Alzheimer’s disease (AD) is relatively novel. To provide clinical references for treating AD, this meta-analysis was performed to evaluate the efficacy and safety of blood derivatives therapy on the patients with AD.

**Methods:**

A systematic articles search was performed for eligible studies published up to December 6, 2021 through the PubMed, Embase, Cochrane library, ClinicalTrials.gov, Chinese National Knowledge Infrastructure database, and Wanfang databases. The included articles were screened by using rigorous inclusion and exclusion criteria. Study selection and data-extraction were performed by two authors independently. Random effects model or fixed effects model was used. Quality of studies and risk of bias were evaluated according to the Cochrane risk of bias tool. All analyses were conducted using Review Manager 5.4. The study was designed and conducted according to the Preferring Reporting Items for Systematic Reviews and Meta-analyses (PRISMA) reporting guideline.

**Results:**

A total of three plasma administrations (two plasma exchange and one young plasma infusion) and five intravenous immunoglobulin (IVIG) randomized controlled trials with a sample size of 1148 subjects diagnosed with AD were included. There was no significant difference in cognitive improvement and all-cause discontinuation between intervention and placebo groups (RR 1.10, 95% CI 0.79–1.54). And Intervention groups showed not a statistically significant improvement in cognition of included subjects measured by the ADAS-Cog (MD 0.36, 95% CI 0.87–1.59), ADCS-ADL (MD −1.34, 95% CI − 5.01–2.32) and NPI (MD 2.20, 95% CI 0.07–4.32) score compared to the control groups. IVIG is well tolerated for AD patients even under the maximum dose (0.4 g/kg), but it is inferior to placebo in Neuropsychiatric Inventory scale in AD patients (MD 2.19, 95% CI 0.02–4.37).

**Conclusions:**

The benefits of blood derivatives therapy for AD are limited. It is necessary to perform well-designed randomized controlled trials with large sample sizes focusing on the appropriate blood derivatives for the specific AD sub-populations in the future.

**Systematic review registration:**

PROSPERO CRD42021233886

**Supplementary Information:**

The online version contains supplementary material available at 10.1186/s13643-022-02115-y.

## Background

As the most common cause of dementia, Alzheimer’s disease (AD) is characterized by progressive cognitive impairment and personality changes [1]. The prevalence of AD is rapidly increasing with the coming acceleration of global population aging, which has been a huge burden on social public expenditure [2–4]. The pathogenesis of AD is complicated and involves widely [5–9]. There are no known effective therapeutics for AD, so it is urgent to seek out a variety of potential treatments that can improve or preserve cognitive function.

Blood derivatives therapy is a conventional treatment that consists of a range of clinical measures, which aims to treat the illness by using blood derivatives such as plasma, blood cells, immunoglobulin, coagulation factor, albumin, and other blood components. Aside from anemia and bleeding, blood derivatives have been utilized in a variety of diseases on account of the diversity of blood components [10, 11]. Currently, the most common blood derivatives therapies for AD are plasma exchange (PE), plasma infusion, and intravenous immunoglobulin (IVIG). PE and young plasma infusion are beneficial in improving the cognitive function of AD patients in some pilot studies [12]. It has been verified that the tissues of the aged mice were rejuvenated and the cognitive function was improved after diluting blood plasma or infusion of young plasma [13, 14]. Although the explicit mechanism is unclear, it may be related to the particular plasma components. IVIG, containing the full range of antibody spectrum, is derived from the plasma of thousands of healthy donors. It has been used to treat patients with autoimmune diseases for decades [15–18]. Moreover, IVIG has been shown to have anti-inflammatory and immunomodulatory functions in addition to anti-amyloid beta (Aβ) [19–21]. On the basis of these benefits, IVIG may be a potential treatment for AD.

At present, there are some randomized controlled trials (RCTs) focusing on the efficacy of blood derivatives therapy for AD, but the obtained conclusions are conflicted [22–24]. In this study, we systematically retrieved the related studies and performed this meta-analysis to evaluate the efficacy of blood derivatives therapy for patients with AD. The safety was also investigated in light of the fact that the majority of AD patients are old and vulnerable to adverse events.

## Methods

This study was designed and conducted according to the Preferred Reporting Items for Systematic Reviews and Meta-analyses (PRISMA) reporting guideline (Additional file 3) [25]. The present protocol has been registered within the PROSPERO database (registration number CRD42021233886).

### Design and search strategy

The search included articles in English or Chinese language published in the PubMed, Embase, Cochrane library, ClinicalTrials.gov, Chinese National Knowledge Infrastructure database, and Wanfang databases through December 6, 2021. The search was conducted using the following keywords: Alzheimer* or cognitive dysfunction or Cognitive Impairment or dementia and blood or plasma or Immunoglobulins, Intravenous or IVIG or Antibodies, Intravenous or transfusion or apheresis or albumin and randomized controlled trial or controlled clinical trial. The detailed retrieval strategy can be found in the supplementary. The references to the included articles and reviews were also searched for citations of additional relevant published and unpublished studies.

### Criteria for inclusion

Inclusion criteria for the systematic review were (1) a randomized controlled study of AD; (2) all subjects were diagnosed with AD; (3) experimental group was not given intervention other than blood derivatives under the guarantee of basic medical care; (4) related scales such as Alzheimer Disease Assessment Scale-Cog (ADAS-cog) were used to evaluate the cognitive function of experimental group and control group before and after the intervention.

### Criteria for exclusion

Studies were excluded if (1) study reported insufficient details to derive the study outcomes; (2) study had other interventions; (3) the full text of the study was not available in the databases; (4) study was written in languages other than English and Chinese.

### Study outcomes

We assessed the primary outcomes of this study including an efficacy measure, improvement in ADAS-cog, which is considered the gold standard for assessing the efficacy of antidementia treatments [26], and a safety measure, all-cause discontinuation. The secondary outcomes including Alzheimer’s Disease Cooperative Study/Activities of Daily Living (ADCS-ADL) [27], Neuropsychiatric Inventory (NPI) [28], the Alzheimer’s Disease Cooperative Study-Clinical Global Impressions of Change (ADCS-CGIC) [29], and Clinical Dementia Rating scale–Sum of Boxes (CDR-sb) [30], were assessed for efficacy. The secondary outcomes for safety were the reported adverse events. We will not restrict the duration of the study and the time point of the evaluation for each outcome.

### Data extraction

Two investigators (FZC, DX) independently performed the literature search and screening, and two investigators (BP, RJP) independently performed data extraction. Discrepancies were resolved through discussion between investigators. The extracted data items include (1) study design, country, study start and end dates, year of publication; (2) participant characteristics, including age, sex, race/ethnicity, size, source; (3) type of dementia, details of the intervention, treatment duration, and all clinical assessment scales.

### Risk of bias

**Fig. 1:**
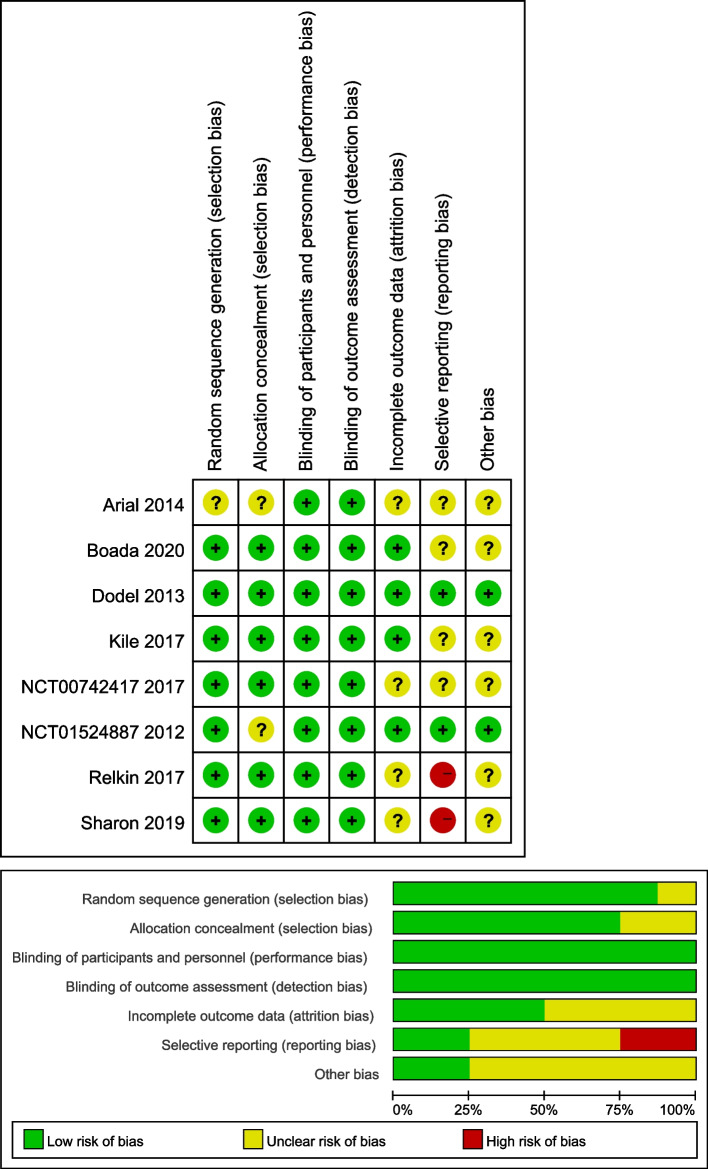
Assessment for risk of bias in included studies

We scored the studies that met inclusion criteria according to the Cochrane risk of bias tool [31], which evaluated the random sequence generation, allocation concealment, blinding of participants, personal and outcome assessment, incomplete outcome data, selective outcome reporting, and other biases (Fig. 1). The included RCTs were classified as low risk (L), high risk (H), or unclear risk (U) in the above items.

### Data synthesis

We summarized the study design and demographic feature of each included study in Table 1. Specifically, information including the year of publication, sample size, drug usage, treatment course, etc. of each individual study will be presented as a summary Table 1.

**Table 1:** Characteristics of RCTs included in this systematic review

Source	Country	Study design	Total, *n*	Subjects intensity	Age (years) mean (SD)	Baseline MMSE mean (SD)	Intervention	Duration	Risk of bias
Merce [12] 2020	Spain and USA	RCT	322	Mild to moderate	69.0 (7.7)	21.6 (2.6)	PE VS placebo	6 weeks + 12 months (6 weeks dosing period and 12 months observation period)	L, L, L, L, U, U
Sha [32] 2019	USA	RCT	18	Mild to moderate	74.2 (3.84)	19.39 (3.24)	Young plasma infusions VS placebo	14 weeks	L, L, L, U, H, U
NCT00742417 [33] 2017	Spain and USA	RCT	37	Mild to moderate	67.7 (7.9)	21.5 (2.8)	PE VS placebo	21 weeks + 6 months (21 weeks dosing period and 6 months observation period)	L, L, L, U, U, U
Kile [34] 2017	USA	RCT	49	MCI	72.3 (7.56)	26.6 (2.4)	IVIG VS placebo	104 weeks (10 weeks dosing period and 94 weeks observation period)	L, L, L, L, U, U
Relkin [23] 2017	USA and Canada	RCT	390	Mild to moderate	70.3 (9.3)	21.3 (3.2)	IVIG VS placebo	78 weeks	L, L, L, U, H, U
NCT01524887 [24] 2012	USA	RCT	261	Mild to moderate	70.8 (9.0)	NR	IVIG VS placebo	78 weeks	L, U, L, L, L, L
Dodel [35] 2013	Germany and USA	RCT	55	Mild to moderate	70.1 (8.2)	21.5 (5.3)	IVIG VS placebo	24 weeks	L, L, L, L, L, L
Arial [36] 2014	Japan	RCT	16	Mild to moderate	72.6	20.0	IVIG VS placebo	26 weeks (12 weeks dosing period and 14 weeks observation period)	U, U, L, U, U, U

*RCT* randomized controlled trial, *PE* plasma exchange, *IVIG* intravenous immunoglobulin, *MCI* mild cognitive impairment, *NR* not reported, *MMSE* Mini-mental State Examination

### Measures of treatment effect

We used 95% confidence intervals (CI) to analyze binary data as risk ratio (RR) and analyzed continuous data as the mean difference (MD), ensuring that the higher scores of consecutive results have the same meaning for a particular result.

### Assessment of heterogeneity

Cochran’s *Q* test and the Higgins *I*^2^ statistic were used to measuring the heterogeneity among the included studies. In addition, we assessed the heterogeneity by visually inspecting the forest plots to assess whether there is a good overlap in CI. The heterogeneity was considered significant if the *I*^2^ is greater than 50%.

### Subgroup and sensitivity analyses

There was no subgroup and sensitivity analysis performed due to the limitation of data. But blood derivatives therapy is divided into plasma administration and IVIG therapy in this meta-analysis.

## Results

**Fig. 2:**
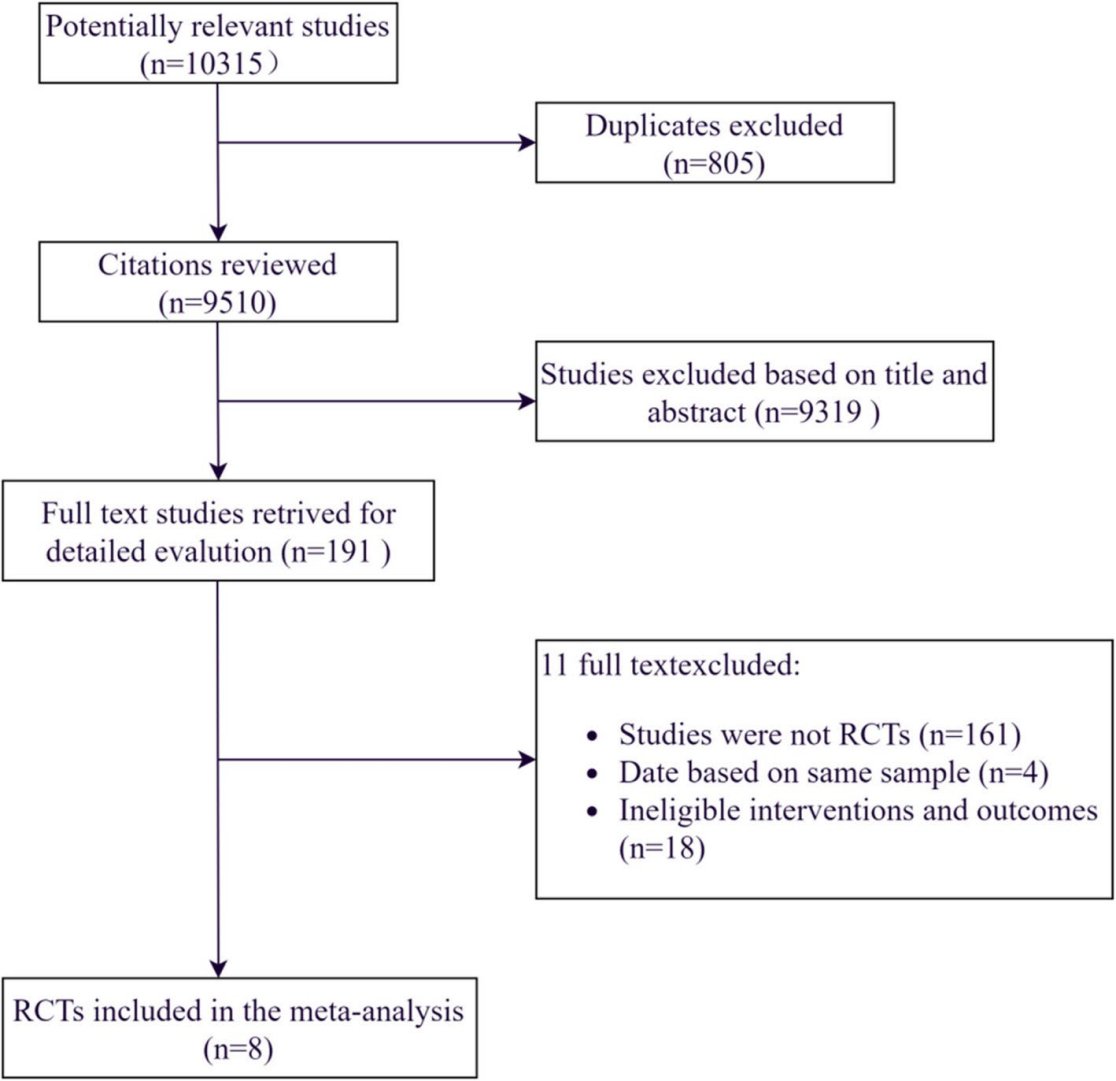
Study flow diagram

A total of 10315 references were identified from the databases (Fig. 2). After excluding duplications and screening of titles and abstracts, the full papers of 191 studies were obtained and assessed for eligibility. According to the inclusion criteria, 8 studies were finally included [12, 23, 24, 32–36]. Plasma-related therapies for AD were summarized as plasma administration in this study. The definitive analysis included 3 plasma administration (2 PE and 1 plasma infusion) RCTs and 5 IVIG RCTs (*n* = 1148) published between 2012 and 2020 for individuals with AD from USA, Spain, Germany, Japan, and Canada. The concrete information of included studies was listed in Table 1. All analyses were conducted using Review Manager 5.4. Random effects model or fixed effects model was performed. There was no sensitivity analysis performed due to the limitation of data.

### Results of the meta-analysis regarding efficacy outcomes

**Fig. 3:**
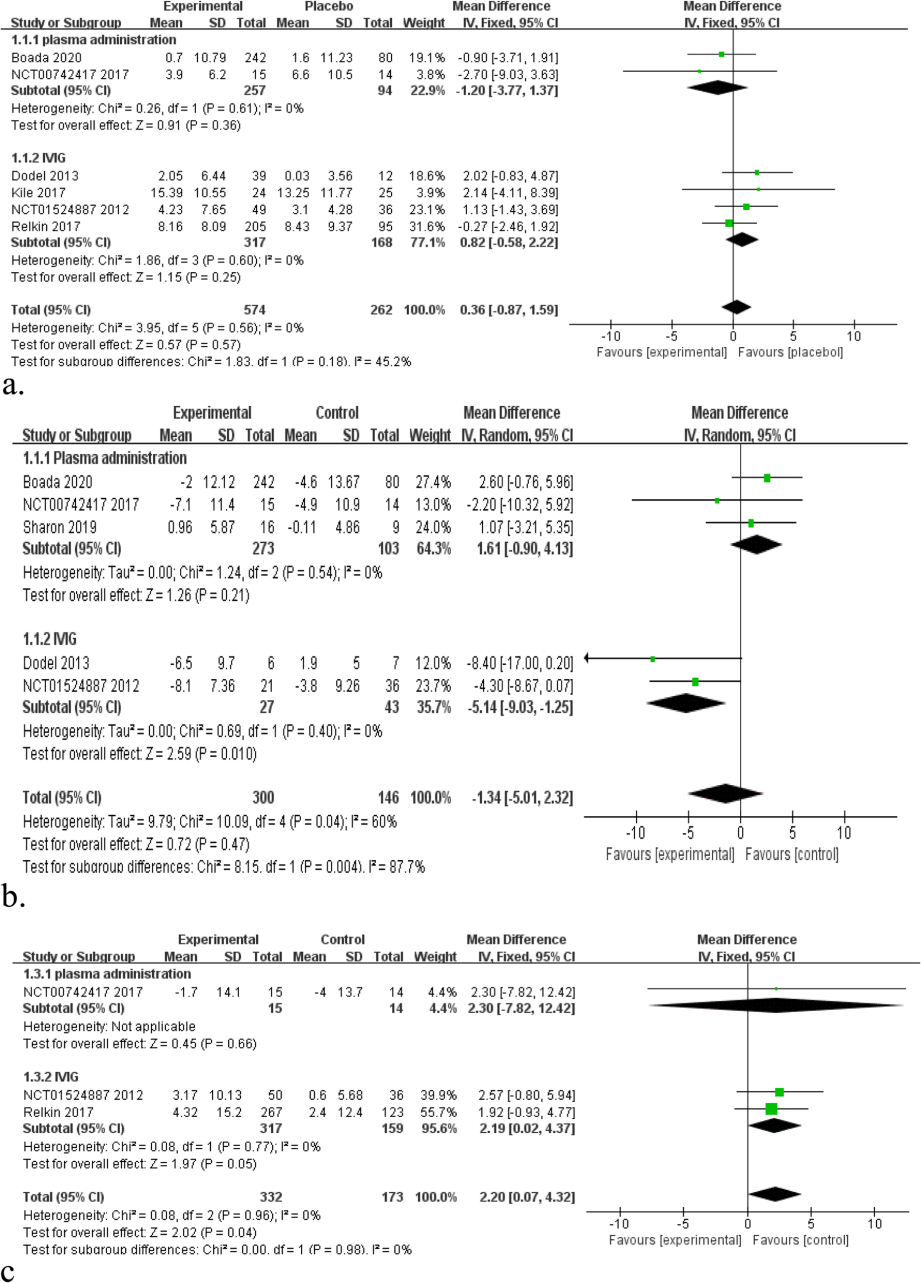
**a** Forest plots of ADAS-Cog scores (6 comparisons, *n* = 836). **b** Forest plots of ADCS-ADL scores (5 comparisons, *n* = 446). **c** Forest plots of NPI scores (3 comparisons, *n* = 505)

Cognitive effects were measured by ADAS-Cog, ADCS-ADL, and NPI in all the included studies. There was no significant difference in ADAS-Cog scores between the intervention (plasma administration and IVIG) and placebo groups (Fig. 3a, MD 0.36, 95% CI 0.87–1.59, *P* = 0.57, *I*^2^ = 0%). For plasma administration groups, 2 RCTs with 351 patients were included in this meta-analysis [12, 33]. The change of ADAS-Cog score was MD − 1.20, 95% CI − 3.77–1.37, *P* = 0.36, *I*^2^ = 0%. It means that plasma administration did not effectively improve the cognition of patients with AD. For IVIG, 4 RCTs with 485 patients were included [23, 24, 34, 35]. The change of ADAS-Cog score was MD 0.82, 95% CI − 0.58–2.22, *P* = 0.25, *I*^2^ = 0%. IVIG also did not achieve satisfactory performance in improving cognitive function.

And there was no significant difference in ADCS-ADL and NPI scores between the intervention (plasma administration and IVIG) and placebo groups (Fig. 3b, MD − 1.34, 95% CI − 5.01–2.32, *P* = 0.47, *I*^2^ = 60%; Fig. 3c, MD 2.20, 95% CI 0.07–4.32, *P* = 0.04, *I*^2^ = 0%). For plasma administration, there was no significant difference in terms of ADCS-ADL (*P* = 0.21) and NPI (*P* = 0.66) scores. It suggested that plasma administration had almost nothing to do with improvement in cognitive function. For IVIG, the maximum dosage of IVIG was superior to placebo group in terms of ADCS-ADL (Fig. 3b): MD − 5.14, 95% CI − 9.03 to − 1.25, *P* = 0.01, *I*^2^ = 0%. However, IVIG was inferior to placebo group in terms of NPI scores (Fig. 3c): MD 2.19, 95% CI 0.02–4.37, *P* = 0.05, *I*^2^ = 0%.

### Results of the meta-analysis regarding safety outcomes

**Fig. 4:**
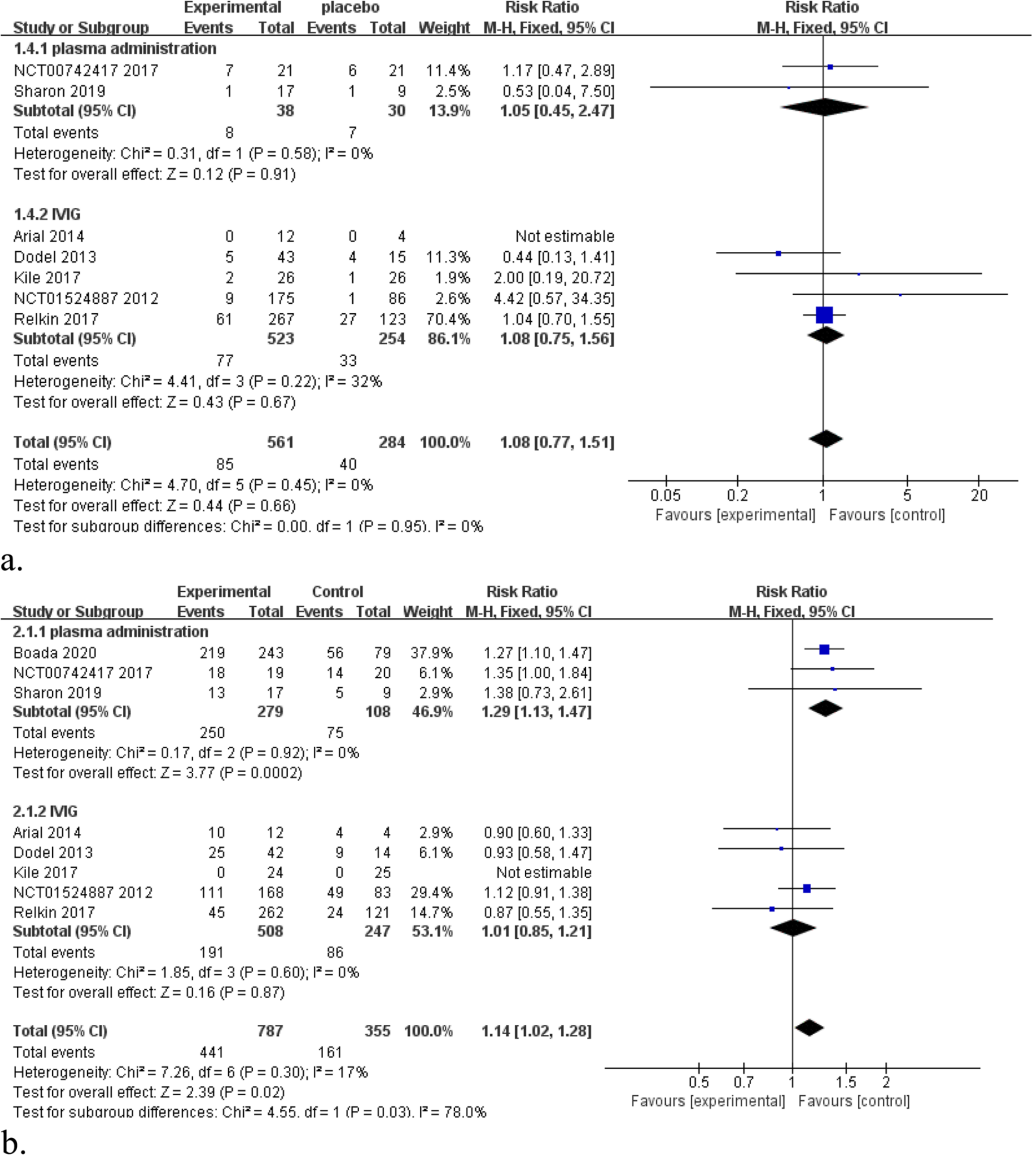
**a** Forest plots of all-cause discontinuation rates (7 comparisons, *n* = 845). **b** Forest plots of adverse events (8 comparisons, *n* = 1142)

All the included studies were performed a safety meta-analysis. It is found that no significant differences between blood derivatives group and placebo group in the number of patients with all-cause discontinuation rates (Fig. 4a, RR 1.10, 95% CI 0.79–1.54, *P* = 0.58, *I*^2^ = 0%). However, plasma administration was associated with a higher incidence of adverse events (Fig. 4b): RR 1.29, 95% CI 1.13–1.47, *P* = 0.0002, *I*^2^ = 0%. There was no difference in adverse events when comparing IVIG with placebo for AD patients (Fig. 4b): RR 1.01, 95% CI 0.85–1.21, *P* = 0.87, *I*^2^ = 0%.

## Discussion

This is the first comprehensive meta-analysis to evaluate the efficacy and safety of blood derivatives therapy for treating AD. In order to ensure the credibility, this meta-analysis only included RCTs with high-quality evidence. All non-randomized controlled trials such as cohort studies were excluded. Similarly, preprints that have not been peer-reviewed will not be included.

For plasma administration group (PE and plasma infusion), the difference from the placebo group in all scale scores did not meet statistical significance. The benefits of plasma administration were only found in limited studies. In Merce 2020 [12], PE was superior to placebo group in terms of CDR-sb and ADCS-CGIC scores, especially for patients with mild AD. And in Sha 2019 [32], young plasma infusion group performed better than placebo group in terms of ADCS-ADL scores.

One of the theoretical basis of PE in the treatment of AD is sink hypothesis [37]. According to the hypothesis, Aβ level is a dynamic equilibrium in cerebrospinal fluid (CSF) and plasma [38]. CSF Aβ level fall when the plasma Aβ are removed by PE, which may be beneficial to AD [39]. In addition, PE may reduce the damage to bodily tissues caused by certain aging factors that may be contained in the blood of the elderly [40]. It has been found that the level of plasma β2 microglobulin (β2M) and C-C motif chemokine 11 (CCL11) rise with age and are positively correlated with the progression of AD [41–45]. PE might provide beneficial effects on AD patients by decreasing β2M, CCL11, and other unknown aging factors. Young plasma infusion may have a beneficial effect on the cognition of elderly recipients by bringing many beneficial factors such as thrombospondin-4, which can promote synaptogenesis and nourish nerves [46]. Young plasma infusions have been successful in mice as the learning and memory function of old mice was considerably improved when they were infused with young plasma [47].

Given these reasons, plasma administration, including PE and plasma infusion, retains therapeutic potential in the treatment of AD. But it needs not only more sophisticated RCTs but also further studies on anti-aging and aging factors in plasma in the future. For safety, plasma administration has a higher incidence of adverse reactions, which may be related to the invasiveness of the treatment and the complexity of plasma component. Although the majority of these adverse events are not lethal and easy to manage such as blood pressure changes, dizziness and infection, care should be taken since older people are more vulnerable to these occurrences.

For IVIG group, the clinical benefits also were limited. It has been demonstrated that IVIG contains anti-Aβ antibody, which can promote the removal of natively formed brain Aβ deposits [48]. However, no significant difference was found between IVIG and placebo treatment groups in terms of ADAS-Cog scores. The high dose (0.4 g/kg) IVIG group achieved better effects only in ADCS-ADL scores (*P* = 0.01). Although the results of this meta-analysis are disappointing, IVIG may improve MCI and brain atrophy in the short term [34]. There was no significant difference in cognitive improvement between IVIG and placebo group after 2 years of intervention, but IVIG alleviated brain atrophy and improved significantly cognitive function in patients with MCI within 1 year [34]. It suggested that IVIG could be more helpful in the treatment of the ultra-early course of AD.

Surprisingly, IVIG group performed the worst in NPI scale, which is often used to evaluate the neurological and mental status of the elderly with dementia, suggesting IVIG deteriorated behavioral and psychological symptoms of AD patients [49]. A possible explanation is that IVIG decreased CSF Aβ levels quickly [23], which is proven to be related to an increase in neuropsychiatric symptoms [50]. Reportedly, the anti-inflammatory cytokine IL-10 showed reverse correlations with total NPI score (the score of 0 is the best in NPI scale) in patients with AD [51], and the presence of IVIG significantly increases the expression of IL-10 in vivo and vitro [52, 53]. But it’s unlikely to apply the higher dose IVIG to increase its anti-inflammatory effects, considering the risk of possible adverse events [54, 55]. AD-specific IVIG may be an alternative approach because it could contain higher concentrations of selected antibodies such as anti-Aβ, anti-tau protein, and anti-inflammatory after purification and recombination and recombinant polyclonal antibody technology [56–60]. Besides, there is growing consensus that cognitive impairment may be due to the neurotoxicity of Aβ oligomers [61]. Therefore, the development of specific IVIG for Aβ oligomers rather than monomers may be another therapeutic option. For safety, there is no significant difference between IVIG and placebo in all-cause discontinuation and adverse events. So, IVIG is a safe treatment.

Since the amyloid cascade hypothesis was published in 1991, AD therapy has focused primarily on monoclonal anti-Aβ antibodies [62], β-secretase inhibitors [63], and γ-secretase modulators [64] and inhibitors [65]. But they did not successfully slow down the progressive loss of cognitive function in patients with AD. The single-target drug may not well interfere with the progression of AD with complex pathogenesis. Plasma is rich in antibody spectrum and beneficial factors, which may be an important resource for searching for drugs against AD. In addition to plasma and IVIG, a new blood derivative GRF6019 has been developed and used in AD research [66]. GRF6019 is made from mixed plasma from healthy donors but depletes immunoglobulins and coagulation factors. It demonstrated excellent safety, feasibility, and tolerability in a pilot trial for the treatment of mild to severe AD patients [67]. Future trials designed to characterize the potential functional benefits of GRF6019 and related plasma fractions in AD are warranted.

## Limitations

Of course, several limitations may affect the results of our meta-analysis. Firstly, although the search strategy is strict, we may not be able to include certain studies, such as non-English or non-Chinese and publications that are not in the searched database. Secondly, the data gathered is limited since this meta-analysis only covers RCTs with high-quality evidence. And the included RCTs were mainly conducted in the USA so the data was only marginally representative. Finally, we cannot exclude the effect of publication bias and the potential effects caused by some confounders.

## Conclusions

Blood derivatives therapy is a safe treatment for AD. But this meta-analysis suggests the benefits of blood derivatives therapy in improving cognition are limited. Due to the complexities of the plasma component and pathogenesis of AD, it is necessary to investigate what plasma components play a role in the treatment of AD. Larger and longer RCTs are needed in the future to confirm the real clinical potential of blood derivatives therapy in AD, especially for MCI patients.

## Supplementary Information

**Additional file 1.**

**Additional file 2.**

**Additional file 3.** PRISMA 2009 Checklist.

**Additional file 4.**

## Data Availability

Not applicable.

## Abbreviations

AD: Alzheimer’s disease
IVIG: Intravenous immunoglobulin
PE: Plasma exchange
Aβ: Amyloid-β
RCTs: Randomized controlled trials
PRISMA: Preferred Reporting Items for Systematic Reviews and Meta-analyses
ADAS-cog: Alzheimer Disease Assessment Scale-Cog
ADCS-ADL: Alzheimer’s Disease Cooperative Study/Activities of Daily Living
NPI: Neuropsychiatric Inventory
ADCS-CGIC: Alzheimer’s Disease Cooperative Study-Clinical Global Impressions of Change
CDR-sb: Clinical Dementia Rating scale–Sum of Boxes
L: Low risk
U: Unclear risk
H: High risk
CSF: Cerebrospinal fluid
β2M: β2 microglobulin
CCL11: C-C motif chemokine 11

## References

[1] Ossenkoppele R, Pijnenburg YA, Perry DC, Cohn-Sheehy BI, Scheltens NM, Vogel JW (2015). The behavioural/dysexecutive variant of Alzheimer’s disease: clinical, neuroimaging and pathological features. Brain.

[2] Alzheimers Association. 2020 Alzheimer's disease facts and figures. Alzheimers Dement. 2020;16(3):391–460.

[3] Long JM, Holtzman DM (2019). Alzheimer disease: an update on pathobiology and treatment strategies. Cell.

[4] Cao H, Du X, Zeng R, Lv Z, Ye S, Jiang P (2019). Effect of different Aβ aggregates as antigen on the measure of naturally occurring autoantibodies against amyloid-β40/42 in IVIG. Curr Alzheimer Res.

[5] Leng F, Edison P (2021). Neuroinflammation and microglial activation in Alzheimer disease: where do we go from here?. Nat Rev Neurol.

[6] Chen Y, Strickland MR, Soranno A, Holtzman DM (2021). Apolipoprotein E: Structural insights and links to alzheimer disease pathogenesis. Neuron.

[7] Hampel H, Vassar R, De Strooper B, Hardy J, Willem M, Singh N (2021). The β-Secretase BACE1 in Alzheimer's Disease. Biol Psychiatry.

[8] John A, Reddy PH (2021). Synaptic basis of Alzheimer's disease: Focus on synaptic amyloid beta, P-tau and mitochondria. Ageing Res Rev.

[9] Ayton S, Bush AI (2021). β-amyloid: The known unknowns. Ageing Res Rev.

[10] Belk JW, Kraeutler MJ, Houck DA, Goodrich JA, Dragoo JL, Mccarty EC (2021). Platelet-rich plasma versus hyaluronic acid for knee osteoarthritis: a systematic review and meta-analysis of randomized controlled trials. Am J Sports Med.

[11] Erdoes G, Koster A, Ortmann E, Meesters MI, Bolliger D, Baryshnikova E (2021). A European consensus statement on the use of four-factor prothrombin complex concentrate for cardiac and non-cardiac surgical patients. Anaesthesia.

[12] Mercè B, López OL, Javier O, Laura N, Michael P, María P (2020). A randomized, controlled clinical trial of plasma exchange with albumin replacement for Alzheimer's disease: Primary results of the AMBAR Study. Alzheimers Dement.

[13] Mehdipour M, Mehdipour T, Skinner CM, Wong N, Liu C, Chen C-C (2021). Plasma dilution improves cognition and attenuates neuroinflammation in old mice. GeroScience.

[14] Conboy IM, Conboy MJ, Wagers AJ, Girma ER, Weissman IL, Rando TAJN (2005). Rejuvenation of aged progenitor cells by exposure to a young systemic environment. Nature.

[15] Henderson LA, Canna SW, Friedman KG, Gorelik M, Lapidus SK, Bassiri H (2021). American College of Rheumatology Clinical Guidance for Multisystem Inflammatory Syndrome in Children Associated With SARS-CoV-2 and Hyperinflammation in Pediatric COVID-19: Version 2. Arthritis Rheum.

[16] Bien CG (2021). Management of autoimmune encephalitis. Curr Opin Neurol.

[17] Dilley M, Wangberg H, Noone J, Geng B (2021). Primary immunodeficiency diseases treated with immunoglobulin and associated comorbidities. Allergy Asthma Proc.

[18] Vani J, Elluru S, Negi V-S, Lacroix-Desmazes S, Kazatchkine MD, Bayary J (2008). Role of natural antibodies in immune homeostasis: IVIg perspective. Autoimmun Rev.

[19] Dubey S, Heinen S, Krantic S, Mclaurin J, Branch DR, Hynynen K (2020). Clinically approved IVIg delivered to the hippocampus with focused ultrasound promotes neurogenesis in a model of Alzheimer's disease. Proc Natl Acad Sci U S A.

[20] Krestova M, Hromadkova L, Bilkova Z, Bartos A, Ricny J (2017). Characterization of isolated tau-reactive antibodies from the IVIG product, plasma of patients with Alzheimer's disease and cognitively normal individuals. J Neuroimmunol.

[21] Lünemann JD, Quast I, Dalakas MCJN (2016). Efficacy of intravenous immunoglobulin in neurological diseases. Neurotherapeutics.

[22] Loeffler DA (2020). AMBAR, an Encouraging Alzheimer's trial that raises questions. Front Neurol.

[23] Relkin NR, Thomas RG, Rissman RA, Brewer JB, Rafii MS, Van Dyck CH (2017). A phase 3 trial of IV immunoglobulin for Alzheimer disease. Neurology.

[24] Nct. Phase 3 IGIV, 10% in Alzheimer’s Disease. https://clinicaltrials.gov/show/NCT01524887, 2012;

[25] Moher D, Liberati A, Tetzlaff J, Altman DG, Medicine PGJP (2009). Preferred reporting items for systematic reviews and meta-analyses: the PRISMA statement. PLoS Med.

[26] Kueper JK, Speechley M, Montero-Odasso M (2018). The Alzheimer's Disease Assessment Scale-Cognitive Subscale (ADAS-Cog): Modifications and Responsiveness in Pre-Dementia Populations. A Narrative Review. J Alzheimers Dis.

[27] Holthoff VA, Marschner K, Scharf M, Steding J, Meyer S, Koch R (2015). Effects of physical activity training in patients with Alzheimer's dementia: results of a pilot RCT study. PLoS One.

[28] Canevelli M, Adali N, Voisin T, Soto ME, Bruno G, Cesari M (2013). Behavioral and psychological subsyndromes in Alzheimer's disease using the Neuropsychiatric Inventory. Int J Geriatr Psychiatry.

[29] Schneider LS, Clark CM, Doody R, Ferris SH, Morris JC, Raman R (2006). ADCS Prevention Instrument Project: ADCS-clinicians' global impression of change scales (ADCS-CGIC), self-rated and study partner-rated versions. Alzheimer Dis Assoc Disord.

[30] Miyagawa T, Brushaber D, Syrjanen J, Kremers W, Fields J, Forsberg LK (2020). Use of the CDR® plus NACC FTLD in mild FTLD: Data from the ARTFL/LEFFTDS consortium. Alzheimers Dement.

[31] Higgins JP, Altman DG, Gøtzsche PC, Jüni P, Moher D, Oxman AD (2011). The Cochrane Collaboration’s tool for assessing risk of bias in randomised trials. BMJ.

[32] Sha SJ, Deutsch GK, Tian L, Richardson K, Coburn M, Gaudioso JL (2019). Safety, tolerability, and feasibility of young plasma infusion in the plasma for Alzheimer symptom amelioration study: a randomized clinical trial. JAMA Neurol.

[33] Nct. Efficacy and safety of plasma exchange with 5% albumin in beta-amyloid peptide clearance in cerebral spinal fluid. https://clinicaltrials.gov/show/NCT00742417, 2017;

[34] Kile S, Au W, Parise C, Rose K, Donnel T, Hankins A (2017). IVIG treatment of mild cognitive impairment due to Alzheimer's disease: a randomised double-blinded exploratory study of the effect on brain atrophy, cognition and conversion to dementia. J Neurol Neurosurg Psychiatry.

[35] Dodel R, Rominger A, Bartenstein P, Barkhof F, Blennow K, Förster S (2013). Intravenous immunoglobulin for treatment of mild-to-moderate Alzheimer's disease: a phase 2, randomised, double-blind, placebo-controlled, dose-finding trial. Lancet Neurol.

[36] Arai H, Ichimiya Y, Shibata N, Nakajima T, Sudoh S, Tokuda T (2014). Safety and tolerability of immune globulin intravenous (human), 10% solution in Japanese subjects with mild to moderate Alzheimer's disease. Psychogeriatrics.

[37] Kheifets V, Braithwaite SP (2019). Plasma-based strategies for therapeutic modulation of brain aging. Neurotherapeutics.

[38] Tapiola T, Alafuzoff I, Herukka S-K, Parkkinen L, Hartikainen P, Soininen H (2009). Cerebrospinal fluid β-amyloid 42 and tau proteins as biomarkers of Alzheimer-type pathologic changes in the brain. Arch Neurol.

[39] Hyman BT, Phelps CH, Beach TG, Bigio EH, Cairns NJ, Carrillo MC (2012). National Institute on Aging–Alzheimer's Association guidelines for the neuropathologic assessment of Alzheimer's disease. Alzheimers Dement.

[40] Rebo J, Mehdipour M, Gathwala R, Causey K, Liu Y, Conboy MJ (2016). A single heterochronic blood exchange reveals rapid inhibition of multiple tissues by old blood. Nat Commun.

[41] Smith LK, He Y, Park J-S, Bieri G, Snethlage CE, Lin K (2015). β2-microglobulin is a systemic pro-aging factor that impairs cognitive function and neurogenesis. Nat Med.

[42] Villeda SA, Luo J, Mosher KI, Zou B, Britschgi M, Bieri G (2011). The ageing systemic milieu negatively regulates neurogenesis and cognitive function. Nature.

[43] Kang JS, Yang YRJA (2020). Circulating plasma factors involved in rejuvenation. Aging (Albany NY).

[44] Bettcher BM, Fitch R, Wynn MJ, Lalli MA, Elofson J, Jastrzab L (2016). MCP-1 and eotaxin-1 selectively and negatively associate with memory in MCI and Alzheimer's disease dementia phenotypes. Alzheimers Dement (Amst).

[45] Parajuli B, Horiuchi H, Mizuno T, Takeuchi H, Suzumura AJG (2015). CCL11 enhances excitotoxic neuronal death by producing reactive oxygen species in microglia. Glia.

[46] Gan KJ, Südhof TC (2019). Specific factors in blood from young but not old mice directly promote synapse formation and NMDA-receptor recruitment. Proc Natl Acad Sci U S A.

[47] Castellano JM, Mosher KI, Abbey RJ, Mcbride AA, James ML, Berdnik D (2017). Human umbilical cord plasma proteins revitalize hippocampal function in aged mice. Nature.

[48] Magga J, Puli L, Pihlaja R, Kanninen K, Neulamaa S, Malm T (2010). Human intravenous immunoglobulin provides protection against Aβ toxicity by multiple mechanisms in a mouse model of Alzheimer's disease. J Neuroinflammation.

[49] Monfort JC, Lezy AM, Papin A, Tezenas Du Montcel S (2020). Psychogeriatric Inventory of Disconcerting Symptoms and Syndromes (PGI-DSS): validity and reliability of a new brief scale compared to the Neuropsychiatric Inventory for Nursing Homes (NPI-NH). Int Psychogeriatr.

[50] Panza F, Lozupone M, Bellomo A, Imbimbo BPJArr. (2019). Do anti-amyloid-β drugs affect neuropsychiatric status in Alzheimer’s disease patients?. Ageing Res Rev.

[51] Holmgren S, Hjorth E, Schultzberg M, Lärksäter M, Frenkel D, Tysen-Bäckström AC (2014). Neuropsychiatric symptoms in dementia—a role for neuroinflammation?. Brain Res Bull.

[52] Kessel A, Ammuri H, Peri R, Pavlotzky ER, Blank M, Shoenfeld Y (2007). Intravenous immunoglobulin therapy affects T regulatory cells by increasing their suppressive function. J Immunol.

[53] Kozicky LK, Zhao ZY, Menzies SC, Fidanza M, Reid GS, Wilhelmsen K (2015). Intravenous immunoglobulin skews macrophages to an anti-inflammatory, IL-10-producing activation state. J Leukoc Biol.

[54] Welles CC, Tambra S, Lafayette RA (2010). Hemoglobinuria and acute kidney injury requiring hemodialysis following intravenous immunoglobulin infusion. Am J Kidney Dis.

[55] Katz U, Achiron A, Sherer Y, Shoenfeld Y (2007). Safety of intravenous immunoglobulin (IVIG) therapy. Autoimmun Rev.

[56] Dodel R, Balakrishnan K, Keyvani K, Deuster O, Neff F, Andrei-Selmer LC (2011). Naturally occurring autoantibodies against beta-amyloid: investigating their role in transgenic animal and in vitro models of Alzheimer's disease. J Neurosci.

[57] Magga J, Puli L, Pihlaja R, Kanninen K, Neulamaa S, Malm T (2010). Human intravenous immunoglobulin provides protection against Aβ toxicity by multiple mechanisms in a mouse model of Alzheimer's disease. J Neuroinflammation.

[58] Du Y, Wei X, Dodel R, Sommer N, Hampel H, Gao F (2003). Human anti-beta-amyloid antibodies block beta-amyloid fibril formation and prevent beta-amyloid-induced neurotoxicity. Brain.

[59] Käsermann F, Boerema DJ, Rüegsegger M, Hofmann A, Wymann S, Zuercher AW (2012). Analysis and functional consequences of increased Fab-sialylation of intravenous immunoglobulin (IVIG) after lectin fractionation. PLoS One.

[60] Taniguchi T, Sumida M, Hiraoka S, Tomoo K, Kakehi T, Minoura K (2005). Effects of different anti-tau antibodies on tau fibrillogenesis: RTA-1 and RTA-2 counteract tau aggregation. FEBS Lett.

[61] Lee SJ, Nam E, Lee HJ, Savelieff MG, Lim MH (2017). Towards an understanding of amyloid-β oligomers: characterization, toxicity mechanisms, and inhibitors. Chem Soc Rev.

[62] Schneider L (2020). A resurrection of aducanumab for Alzheimer's disease. Lancet Neurol.

[63] Moussa-Pacha NM, Abdin SM, Omar HA, Alniss H, Al-Tel TH (2020). BACE1 inhibitors: Current status and future directions in treating Alzheimer's disease. Med Res Rev.

[64] Lessard CB, Rodriguez E, Ladd TB, Minter LM, Osborne BA, Miele L (2020). γ-Secretase modulators exhibit selectivity for modulation of APP cleavage but inverse γ-secretase modulators do not. Alzheimers Res Ther.

[65] Yang G, Zhou R, Guo X, Yan C, Lei J, Shi Y (2021). Structural basis of γ-secretase inhibition and modulation by small molecule drugs. Cell.

[66] Hannestad J, Koborsi K, Klutzaritz V, Chao W, Ray R, Páez A (2020). Safety and tolerability of GRF6019 in mild-to-moderate Alzheimer's disease dementia. Alzheimers Dement (N Y).

[67] Hannestad J, Duclos T, Chao W, Koborsi K, Klutzaritz V, Beck B (2021). Safety and tolerability of GRF6019 infusions in severe Alzheimer's disease: a phase II double-blind placebo-controlled trial. J Alzheimers Dis.

